# Vehicle identification from automotive paints transferred in road accidents

**DOI:** 10.1080/07853890.2021.1897431

**Published:** 2021-09-28

**Authors:** Sandra Coelho, M. Catarina de Almeida, Carlos Família

**Affiliations:** a Instituto Universitário Egas Moniz (IUEM), Egas Moniz Cooperativa de Ensino Superior, Caparica, Portugal; b Centro de Investigação Interdisciplinar Egas Moniz (CiiEM), Egas Moniz Cooperativa de Ensino Superior, Caparica, Portugal

## Abstract

**Introduction:**

Paint chips and smears represent the most common types of trace evidence resulting from vehicle-related incidents [1,2]. Analysis of these traces would ideally lead to the identification of the paint producer and ultimately of the vehicle in question [2]. Despite the chemical complexity of automotive paints, several reports showed this identification to be possible through the use Py-GC/MS, SEM-EDX, WDXRF, Raman and FTIR spectroscopy [1,3–5]. However, these studies were focussed on the analysis of each of the individual layers that compose the painting, making the analysis more complex, time consuming and dependent on highly specialised equipment. In the present work, we evaluated the discriminative potential of ATR-FTIR for the identification of paint producers, based on the analysis of the paint as a whole.

**Materials and methods:**

Fragments of 6 different car paints obtained from the hood and lateral panels were milled and analysed through ATR-FTIR, both individually and as mixtures between pairs of paint Figure 1. Spectra were obtained between 450 and 4000 cm^−1^, with a resolution of 4 cm^−1^ and 16 accumulations.

**Results:**

The obtained results show the paint spectra to have considerable differences both in the number and intensity of IR bands. Furthermore, the mixtures’ spectra clearly show bands of both individual paints that are proportional, in intensity, to the ratio of each individual paint. **Discussion and conclusions:** These preliminary results show considerable differences between the FTIR spectra of the individual paints that propagate throughout the paint mixtures. This suggests that there are features in the ATR-FTIR spectra of automotive paints, as a whole that might be suitable predictors for the development of highly accurate predictive models.

**Figure 1.**
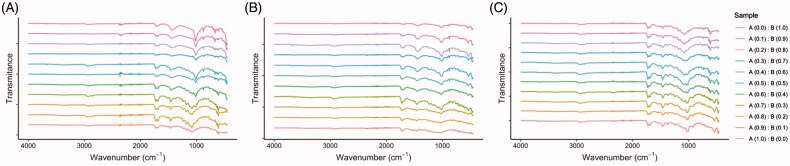
ATR-FTIR spectra of the isolated paints (top and bottom spectra), and of the mixtures at different ratios (middle spectra), where A) corresponds to a blue Volvo (A) and a red Renault (B); (B) to a blue Citröen (A) and a grey Skoda (B); (C) to a black Peugeot (A) and a grey Seat (B).
